# Proton radiotherapy as a treatment strategy to increase survival in locally advanced pancreatic cancer in the body and tail: a retrospective study

**DOI:** 10.1186/s13014-023-02301-9

**Published:** 2023-08-08

**Authors:** Katsuya Ami, Kazuki Terashima, Jun Ishida, Masaki Suga, Taisuke Okawa, Daiki Takahashi, SungChul Park, Yoshiro Matsuo, Yoshihide Nanno, Sunao Tokumaru, Tomoaki Okimoto, Hirochika Toyama, Takumi Fukumoto

**Affiliations:** 1 Division of Hepato-Biliary-Pancreatic Surgery, Department of Surgery, Kobe University Graduate School of Medicine, 7-5-2 Kusunoki-cho, Chuo-ku, Kobe, Hyogo 650-0017 Japan; 2 Department of Radiology, Hyogo Ion Beam Medical Center, 1-2-1 Kouto, Shingu-cho, Tatsuno, Hyogo 679-5165 Japan; 3 Department of Radiation Physics, Hyogo Ion Beam Medical Center, 1-2-1 Kouto, Shingu-cho, Tatsuno, Hyogo 679-5165 Japan

**Keywords:** Pancreatic cancer in the body and tail, Locally advanced pancreatic cancer, Proton radiotherapy, Chemoradiotherapy, Multidisciplinary treatment, Prognostic factor, Long-term survival outcome, Retrospective study

## Abstract

**Background:**

Long-term outcomes and prognostic factors of proton radiotherapy for locally advanced pancreatic cancer (LAPC) in the body and tail are still unknown. The aim of this study was to determine the prognostic factors after proton radiotherapy in a large group of patients with LAPC in the body and tail.

**Methods:**

The medical records of 200 patients with LAPC in the body and tail who underwent proton radiotherapy between February 2009 and January 2021 at the Hyogo Ion Beam Medical Center were retrospectively reviewed to identify prognostic factors that contribute to long-term survival.

**Results:**

The overall survival rate at 1- and 2-year after PT was 69.6% and 35.4% with a median overall survival of 18.4 months. The 1- and 2-year local progression-free, and progression-free survival rates were 84.3% and 68.0%, and 44.3% and 19.4%, respectively. In multivariate analysis, superior mesenteric artery (SMA) invasion (SMA only invasion vs. celiac artery only invasion; *P* = 0.049: SMA and celiac artery invasion vs. celiac artery only invasion; *P* = 0.017), carbohydrate antigen 19-9 (CA 19-9) level ≥ 231.9 U/mL (*P* = 0.001), anterior peripancreatic invasion (*P* = 0.006), and incomplete scheduled concurrent chemotherapy (*P* = 0.009) were statistically significant prognostic factors for overall survival. There was no significant difference in local progression-free survival; however, distant metastasis-free survival was statistically worse in patients with prognostic factors than in those without.

**Conclusions:**

Proton radiotherapy for LAPC in the body and tail may be a valuable multidisciplinary treatment option. Patients with SMA invasion, higher pre-proton radiotherapy serum CA 19-9 level, anterior peripancreatic invasion, or incomplete scheduled concurrent chemotherapy had worse overall survival because of worse distant metastasis-free survival, suggesting that distant metastases have a significant impact on overall survival in such patients.

*Trial registration*: Retrospectively registered.

## Background

Pancreatic cancer is the fourth leading cause of cancer-related deaths, with an estimated 5-year survival rate of 9% [1]. Although surgical resection is a potentially curative treatment, only 10–15% of patients have resectable disease at diagnosis. While approximately 50% of patients present with distant metastatic disease, more than 30% are diagnosed with unresectable pancreatic cancer due to local invasion without distant metastasis [locally advanced pancreatic cancer (LAPC)] [2]. Chemotherapy or chemoradiotherapy is historically considered the standard therapy for LAPC [3], with unclear superiority. Although pancreatic cancer is considered a systemic disease, previous studies have shown that 30–40% of patients with LAPC die of local progression without distant metastases [4, 5]. In addition, LAPC may be symptomatic if it involves adjacent structures such as the abdominal nerve plexus or bile duct. Local control with radiotherapy for primary tumors may provide survival and palliative benefits in the treatment of LAPC.

Recently, particle radiotherapy, such as proton and carbon ion radiotherapy, has become increasingly widespread across the world, the commissioned indications being regularly reviewed in several health care services as new evidence emerges [6, 7]. However, capacity is still limited and access is not equitable globally. Particle radiotherapy is characterized by the Bragg peak phenomenon and can cover the tumor volume with high accuracy because the doses to the surrounding normal tissue are effectively reduced; the first big prospective series showing promising outcomes in both cranial and extracranial settings [8–10]. Particle radiotherapy for LAPC has attracted attention because of the proximity of the pancreas to the radiation-sensitive gastrointestinal tract.

In 2012, we published the world’s first report on the treatment outcome of gemcitabine-concurrent proton radiotherapy (PT) for LAPC in a phase I/II trial [11]. Other reports of PT for LAPC demonstrated that the 1- and 2-year overall survival (OS) rates were 61–73% and 31–46%, respectively [12, 13]. Kawashiro et al. reported that the median survival time and 1- and 2-year OS rates after carbon ion therapy for LAPC were 21.5 months, 73%, and 46%, respectively [14]. Recently, we identified the long-term outcomes of gemcitabine-concurrent PT for LAPC in 123 patients [15]. In this study, it was suggested that patients with LAPC in the body and tail had longer survival than those with LAPC in the head. LAPC in the body and tail may be more suitable for PT because irradiation doses for LAPCs in the head are consistently restricted as they are frequently adjacent to the second or third portions of the duodenum. Clinical treatment differs owing to differences not only in the irradiation doses but also in follow-up and complications between LAPC in the head, which has more bile duct invasion, and LAPC in the body and tail, which has less bile duct invasion; thus, these two should be clinically distinguished. Therefore, we conducted a retrospective analysis to evaluate PT in a larger group of patients with LAPC in the body and tail. This study aimed to examine the safety and prognosis of PT to identify prognostic factors that contribute to long-term survival.

## Methods

### Patients

The medical records of patients with LAPC in the body and tail (n = 200) who underwent PT (67.5 GyE in 25 fractions) between February 2009 and January 2021 at the Hyogo Ion Beam Medical Center were retrospectively reviewed. This study was approved by the Institutional Review Board of Hyogo Ion Beam Medical Center (Approval # 5-1) and complied with the Declaration of Helsinki; the need for informed consent was waived owing to the retrospective nature of the study. The diagnosis of pancreatic cancer was confirmed histologically or clinically by tumor markers and diagnostic imaging, such as computed tomography (CT) and magnetic resonance imaging. For the pretreatment evaluation, all patients underwent abdominal and chest contrast-enhanced CT as well as positron emission tomography (PET) with 18F-fluorodeoxyglucose (FDG) to exclude distant metastasis and gastroscopy for exclude gastrointestinal mucosal invasion. The presence of tumor invasion of surrounding organs such as the bile duct, duodenum, vessels, extrapancreatic nerve plexus, and other organs was determined by radiological density anomalies in contrast-enhanced CT.

### Proton radiotherapy

Patients were treated with 150–210 MeV proton beams. CT without an intravenous contrast agent were taken during the expiratory phase under a respiratory gating system prior to finalization of treatment plans. Patients were immobilized in the prone position using a custom-made thermoplastic cast, and the setup was performed daily by subtraction of the two sets of orthogonal digital radiographs before each irradiation using bony landmarks and one fiducial marker attached to a branch of the gastroduodenal and/or dorsal pancreatic arteries on angiography.

Treatment plans were developed using a 2-mm slice thickness CT-based three-dimensional treatment planning system (Mitsubishi Electric, Tokyo, Japan). The gross tumor volume (GTV) included the primary tumor plus the apparently involved lymph nodes, as determined by a fusion contrast-enhanced CT subsidiary using FDG-PET. The clinical target volume comprised the addition of a 5-mm margin to the GTV and prophylactic irradiation regions containing draining lymph nodes, para-aortic lymph nodes, and peripheral regions around the celiac artery (CA) and superior mesenteric artery (SMA), excluding the gastrointestinal tract. The planning target volume was defined as the plus setup margin (5 mm) and a respiratory gating margin (1–5 mm), which were measured on CT images between the inspiratory and expiratory phases. The relative biological effectiveness of the treatment beam was determined to be 1.1 [16]. The total dose of 67.5 GyE was divided into 25 daily fractions using the field-in-field technique [11]. Generally, the stomach, duodenum, small intestine, kidneys, and spinal cord, are defined as organs at risk. Dose restrictions for the stomach, duodenum, and spinal cord were approximately 50, 50, and 45 GyE, respectively.

### Concurrent chemotherapy

Concurrent chemotherapy was administered using gemcitabine or S-1 (tegafur/gimeracil/oteracil) monotherapy, if feasible. Gemcitabine (GEM; 800 mg/m^2^) was administered via intravenous infusion for the initial 3 weeks of the 5-week PT period. S-1 was administered at a dose of 80 mg/m^2^ twice daily on the day of PT irradiation. If the patient was not fit for chemotherapy due to age or poor performance status, PT was administered without concurrent chemotherapy.

### Follow-up

After PT, all patients underwent repeated contrast-enhanced CT and/or FDG-PET scans and tumor marker monitoring every 3 months. We defined local progression as radiographic enlargement of the primary tumor, locoregional recurrence, or elevation of tumor markers including carcinoembryonic antigen (CEA) and carbohydrate antigen 19-9 (CA19-9) for at least 3 months without any distant metastases. Toxicity was defined and graded using the National Cancer Institute Common Terminology Criteria for Adverse Events (version 5.0). Toxicities were classified into two categories according to the time of onset: (1) early: within three months after PT and (2) late: later than three months after PT. All patients underwent gastrointestinal endoscopy before proton therapy and every 3–6 months after therapy.

### Statistical analysis

The patient demographics and treatment characteristics were summarized using descriptive statistics. Continuous variables are expressed as medians (ranges). Kaplan–Meier curves were used to estimate survival outcomes, such as OS, progression-free survival (PFS), local progression-free survival (LPFS), and distant metastasis-free survival (DMFS). Log-rank tests and Cox regression models were used for univariate and multivariate analyses to investigate the prognostic factors for OS. A *P* value < 0.05 was considered significant in all statistical analyses, which were performed using JMP 16 (SAS Institute Japan, Tokyo, Japan).

## Results

### Patient and treatment characteristics

Patient characteristics are shown in Table 1. This study investigated 200 patients (91 women and 109 men) with a median age of 65 years (38–88 years). The median tumor size was 36 mm (15–70 mm). The median CA19-9 and CEA levels were 231.9 U/mL (0.1–19,500) and 3.5 ng/mL (0.5–135.1), respectively. Ninety-one (45.5%), 37 (18.5%), and 72 (36.0%) patients had CA, SMA, and both CA and SMA invasion, respectively. CT images obtained before irradiation showed anterior peripancreatic invasion in 130 patients (65.0%). All patients underwent irradiation PT with 67.5 GyE in 25 fractions and completed the planned treatment. The median GTV was 46.3 cm^3^ (4.7–205.6), and the volume ratio irradiated over 60 GyE at the GTV (GTV V60GyE) was 64.2% (25.2–100%). Concurrent chemotherapy was planned for 186 patients (93.0%). Of those, 140 patients (75.3%, gemcitabine, n = 123, S1, n = 17) completed the planned chemotherapy regimen (Table 1). Of the 14 patients (7.0%) for whom concurrent chemotherapy was not planned, 9 patients refused chemotherapy owing to the side effects of previous chemotherapy, 3 patients were elderly, and 2 patients had an Eastern Cooperative Oncology Group-Performance Status of 2 points.

**Table 1:** Patient characteristics

Characteristics	All patients n = 200 (range or %)
Age, years	65 (38–88)
Gender	
Male	109 (54.5)
Female	91 (45.5)
Body mass index, kg/m^2^	20.1 (13.2–29.0)
ECOG-PS	
0	141 (70.5)
1	57 (28.5)
2	2 (1.0)
CA19-9, U/mL	231.9 (0.1–19,500)
CEA, ng/mL	3.5 (0.5–135.1)
Pathological diagnosis	
Yes	132 (66.0)
No	68 (34.0)
Tumor size, mm	36 (15–70)
Major arterial invasion	
CA	91 (45.5)
SMA	37 (18.5)
CA and SMA	72 (36.0)
UICC T classification	
1–3	0 (0)
4	200 (100)
UICC N classification	
0	137 (68.5)
1	60 (30.0)
2	3 (1.5)
UICC stage	
I, II	0 (0)
III	200 (100)
Bile duct invasion	
Positive	30 (15.0)
Negative	170 (85.0)
Duodenal invasion	
Positive	18 (9.0)
Negative	182 (91.0)
Anterior peripancreatic invasion	
Positive	130 (65.0)
Negative	70 (35.0)
Posterior peripancreatic invasion	
Positive	200 (100)
Negative	0 (0)
Venous invasion	
Positive	182 (91.0)
Negative	18 (9.0)
Arterial invasion	
Positive	200 (100)
Negative	0 (0)
Extrapancreatic nerve plexus invasion	
Positive	200 (100)
Negative	0 (0)
Other organ invasion	
Positive	9 (4.5)
Negative	191 (95.5)
Previous treatment	
Yes	106 (53.0)
FOLFIRINOX	6 (3.0)
GnP	16 (8.0)
FOLFIRINOX and GnP	51 (25.5)
Others	33 (16.5)
No	94 (47.0)
Complete scheduled concurrent chemotherapy	
Yes	140 (70.0)
GEM	123 (61.5)
S1	17 (8.5)
No	60 (30.0)
Incomplete GEM	36 (18.0)
Incomplete S1	10 (5.0)
No concurrent chemotherapy	14 (7.0)
Median GTV volume, cc	46.3 (4.7–205.6)
Median CTV volume, cc	189.6 (53.3–424.0)
Median GTV V60GyE, %	64.2 (25.2–100)
Median CTV V60GyE, %	61.2 (30.3–97.3)

*ECOG-PS* Eastern Cooperative Oncology Group-Performance Status, *CA* coeliac artery, *SMA* superior mesenteric artery, *UICC* Union for International Cancer Control, *TNM* tumor-node-metastasis classification 8th edition, *CEA* carcinoembryonic antigen, *CA19-9* carbohydrate antigen 19-9, *GnP* gemcitabine and nab-paclitaxel, *GEM* gemcitabine, *S-1* tegafur/gimeracil/oteracil, *GTV* gross tumor volume, *CTV* clinical target volume, *GyE* Gy equivalents, *V60GyE* the volume ratio irradiated over 60 GyE

### Toxicity

Acute and late toxicities are summarized in Table 2. Acute grade ≥ 3 hematologic toxicity was observed in 71 patients (35.5%, leukocytopenia, n = 66, thrombocytopenia, n = 5) (Table 2). Grade 3 toxicity, including gastrointestinal bleeding/ulceration, nausea/vomiting, anorexia, and dermatitis, was observed in 15 (7.5%), 3 (1.5%), 2 (1.0%), and 2 (1.0%) patients, respectively. Late grade 3 gastrointestinal bleeding/ulcer was observed in 14 patients (7.0%), and there was one case (0.5%) of grade 5 toxicity.

**Table 2:** Acute and late toxicities in all grades of patients who underwent proton radiotherapy

Toxicity	Grade 1/2 n (%)	Grade 3/4 n (%)	Grade 5 n (%)	All n (%)
Acute toxicities				
Hematologic				
Leukocytopenia	68 (34.0)	66 (33.0)	–	134 (67.0)
Thrombocytopenia	26 (13.0)	5 (2.5)	–	31 (15.5)
Gastrointestinal				
Gastrointestinal bleeding/ulcer	46 (23.0)	15 (7.5)	–	61 (30.5)
Nausea/vomiting	12 (6.0)	3 (1.5)	–	15 (7.5)
Diarrhea	4 (2.0)	–	–	
Anorexia	16 (8.0)	2 (1.0)	–	18 (9.0)
Others				
Dermatitis	40 (20.0)	2 (1.0)	–	42 (21.0)
Jaundice	2 (1.0)	–	–	2 (1.0)
Late toxicities				
Gastrointestinal				
Gastrointestinal bleeding/ulcer	43 (19.5)	14 (7.0)	1 (0.5)	58 (29.0)
Anorexia	2 (1.0)	–	–	2 (1.0)
Others				
Dermatitis	14 (7.0)	–	–	14 (7.0)
Spinal fracture	2 (1.0)	–	–	2 (1.0)

### Patient survival and prognostic factors

**Fig. 1:**
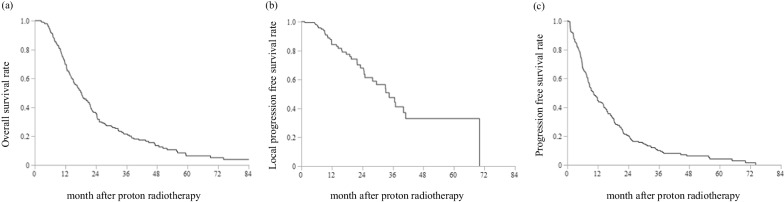
Survival curves after proton radiotherapy for all patients. **a** Overall survival, **b** local control rate, and **c** progression-free survival

The median follow-up time was 15.2 months (1.2–118.8). The OS rate at 1- and 2-year after PT was 69.6% and 35.4%, respectively, with a median overall survival (mOS) of 18.4 months (95% confidence interval [CI] 15.2–21.5) (Fig. 1a). The median overall survival (mOS) after PT was 18.4 months (95% CI 15.2–21.5) (Fig. 1a). The 1- and 2-year OS rates were 69.6% and 35.4%, respectively. The 1- and 2-year LPFS rates were 84.3% and 68.0% (Fig. 1b), while the 1- and 2-year PFS rates were 44.3% and 19.4%, respectively (Fig. 1c).

**Fig. 2:**
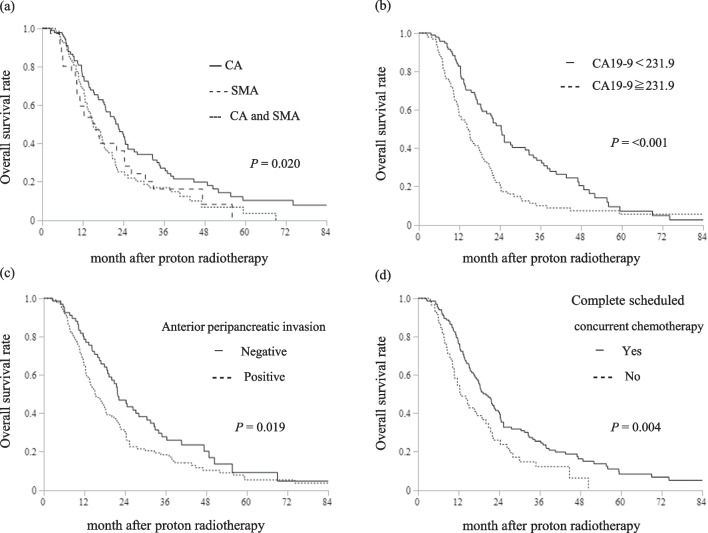
Survival curves after proton radiotherapy for subsets of patients. **a** Overall survival curves of patients with locally advanced pancreatic cancer (LAPC) at the major arterial invasion, CA celiac artery; SMA superior mesenteric artery; and CA and SMA. **b** Overall survival curves of patients with LAPC CA19-9 ≧ 231.9 or CA19-9 < 231.9. **c** Overall survival curves of patients with LAPC with and without anterior peripancreatic invasion. **d** Overall survival curves of patients with LAPC with and without complete scheduled concurrent chemotherapy

**Fig. 3:**
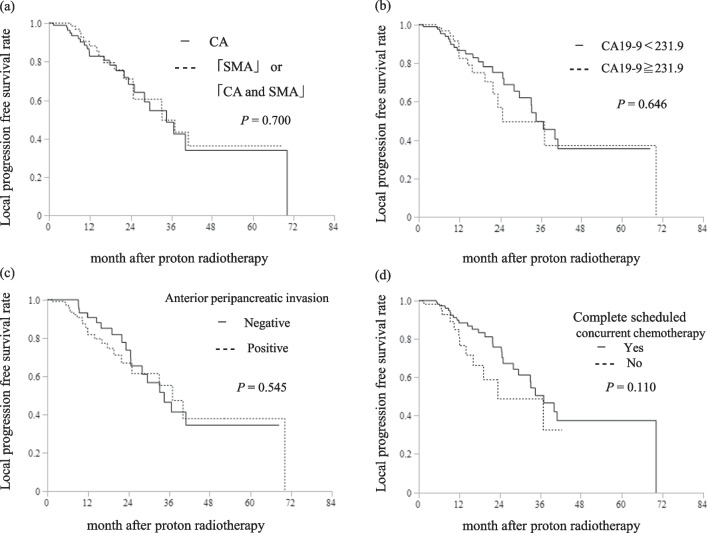
Survival curves after proton radiotherapy for subsets of patients. **a** Local progression free survival curves of patients with locally advanced pancreatic cancer (LAPC) at the major arterial invasion, CA celiac artery; SMA superior mesenteric artery; and CA and SMA. **b** Local progression free survival curves of patients with LAPC CA19-9 ≧ 231.9 or CA19-9 < 231.9. **c** Local progression free survival curves of patients with LAPC with and without anterior peripancreatic invasion. **d** Local progression free survival curves of patients with LAPC with and without complete scheduled concurrent chemotherapy

**Fig. 4:**
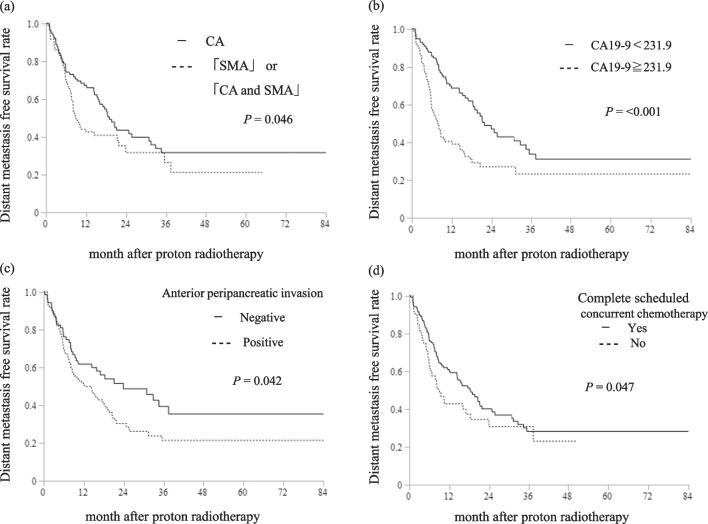
Survival curves after proton radiotherapy for subsets of patients. **a** Distant metastasis free survival curves of patients with locally advanced pancreatic cancer (LAPC) at the major arterial invasion, CA celiac artery; SMA superior mesenteric artery; and CA and SMA. **b** Distant metastasis free survival curves of patients with LAPC CA19-9 ≧ 231.9 or CA19-9 < 231.9. **c** Distant metastasis free survival curves of patients with LAPC with and without anterior peripancreatic invasion. **d** Distant metastasis free survival curves of patients with LAPC with and without complete scheduled concurrent chemotherapy

In the univariate analyses, body mass index < 18.5 kg/m^2^, pre-PT serum CA 19-9 level > 231.9 U/mL, CEA level > 3.5 ng/mL, SMA invasion (SMA only vs. CA only, CA and SMA vs. CA only), anterior peripancreatic invasion, and incomplete scheduled concurrent chemotherapy were associated with a shorter OS (Table 3). In multivariate analysis, CA 19-9 ≥ 231.9 U/mL (hazard ratio [HR]: 1.75, 95% CI 1.27–2.43, *P* = 0.001), SMA invasion (HR [SMA only invasion vs. CA only invasion]: 1.62, 95% CI 1.00–2.62, *P* = 0.049. HR [CA and SMA invasion vs. CA only invasion]: 1.56, 95% CI 1.08–2.25, *P* = 0.017), anterior peripancreatic invasion (HR: 1.65, 95% CI 1.15–2.43, *P* = 0.006), and incomplete scheduled concurrent chemotherapy (HR: 1.63, 95% CI 1.13–2.35, *P* = 0.009) were statistically significant prognostic factors for OS (Table 3). Figure 2 shows the Kaplan–Meier curves of OS in each subset of patients with and without these prognostic factors. There was no significant difference in the LPFS between patients with and without these prognostic factors (Fig. 3). Patients with these prognostic factors had significantly worse DMFS than those without (Fig. 4).

**Table 3:** Univariate and multivariate analyses of prognostic factors for overall survival (n = 200)

Variables	Univariate		Multivariate	
	HR (95% CI)	*P* value	HR (95% CI)	*P* value
Age ≧ 65 years	1.06 (0.65–1.73)	0.822		
Gender, Male	0.97 (0.71–1.32)	0.836		
Body mass index < 18.5 kg/m^2^	1.43 (1.01–2.01)	**0.044**	1.17 (0.81–1.68)	0.403
ECOG-PS, 1–2	1.04 (0.75–1.46)	0.811		
CA19-9 ≧ 231.9 U/mL	1.86 (1.35–2.55)	**< 0.001**	1.75 (1.27–2.43)	**0.001**
CEA ≧ 3.5 ng/mL	1.39 (1.02–1.91)	**0.040**	1.12 (0.81–1.55)	0.502
Previous treatment, Yes	1.26 (0.92–1.73)	0.149		
Major arterial invasion				
SMA only versus CA only	1.61 (1.03–2.50)	**0.036**	1.62 (1.00–2.62)	**0.049**
SMA and CA versus CA only	1.56 (1.10–2.22)	**0.014**	1.56 (1.08–2.25)	**0.017**
Tumor size ≧ 40 mm	1.01 (0.73–1.37)	0.963		
Bile duct invasion, positive	1.24 (0.81–1.91)	0.322		
Duodenal invasion, positive	1.44 (0.85–2.44)	0.170		
Anterior peripancreatic invasion, positive	1.49 (1.07–2.09)	**0.020**	1.65 (1.15–2.43)	**0.006**
Venous invasion, positive	1.25 (0.70–2.20)	0.450		
Other organ invasion, positive	1.15(0.47–2.82)	0.755		
UICC N, 1 and 2	0.94 (0.67–1.32)	0.742		
Complete scheduled concurrent chemotherapy, No	1.66 (1.17–2.34)	**0.004**	1.63 (1.13–2.35)	**0.009**
Acute toxicities CTCAE grade ≧ 3	1.20 (0.87–1.65)	0.259		
Late toxicities CTCAE grade ≧ 3	1.43 (0.81–2.53)	0.220		
GTV V60 GyE ≧ 60%	0.98 (0.71–1.35)	0.911		

*HR* hazard ratio, *CI* confidence interval, *ECOG-PS* Eastern Cooperative Oncology Group-Performance Status, *CEA* carcinoembryonic antigen, *CA19-9* carbohydrate antigen 19–9, *SMA* superior mesenteric artery, *CA* coeliac artery, *UICC* Union for International Cancer Control, *TNM* tumor-node-metastasis classification 8th edition, *CTCAE* common terminology criteria for adverse events, *GTV* gross tumor volume, *GyE* Gy equivalents, *V60GyE* the volume ratio irradiated over 60 GyE

Significant *P* values (< 0.05) are in bold

## Discussion

In the present study, the mOS, 1- and 2-year OS rates, and 1- and 2-year LPFS rates after PT for LAPC in the body and tail were 18.4 months, 69.6%, and 35.4%, 84.3%, and 68.0%, respectively. This is comparable to other intensive chemotherapy regimens, such as gemcitabine and nab-paclitaxel (GnP) and FOLFIRINOX (mOSs, 18.8–21.2 and 14.0–24.2 months, respectively) [17–19] and to chemoradiotherapy (mOSs, 15.7–21.4 months) [20–23]. Multivariate Cox regression analysis revealed that SMA invasion, high pre-PT serum CA 19-9, anterior peripancreatic invasion, and incomplete scheduled concurrent chemotherapy were independent prognostic factors. To the best of our knowledge, this is the largest study to investigate short- and long-term outcomes after PT for LAPC.

Regarding the identified prognostic factors (SMA invasion, anterior peripancreatic invasion, and higher pre-PT serum CA 19-9), there were no significant differences in LPFS between patients with and without these factors. However, patients with these factors had significantly worse DMFS than those without, which may have contributed to worse survival due to distant metastases after PT. The drawbacks of PT include the possibility of overlooking radiologically negative distant metastases that can be detected by surgical exploration; thus, it is important to rule out distant metastases rigorously before PT. Gadoxetic acid-enhanced magnetic resonance imaging is reportedly effective in detecting liver metastases [24]. Recent studies have shown that staging laparoscopy is useful for diagnosing radiologically negative distant metastases [25–28]. Such pretherapeutic management may lead to better patient selection and prolonged survival after PT.

In the context of controlling occult metastases, peri-PT chemotherapy may have oncological benefits. In the present study, patients with complete scheduled concurrent chemotherapy in which GEM or S-1 was used had significantly longer DMFS and OS than those without it. GEM or S-1 has broad-spectrum antitumor activity against a variety of solid tumors and acts as a potent radiosensitizer in pancreatic cancer [29, 30]. However, some patients are unable to receive concurrent chemotherapy because of poor performance status or severe side effects of previous chemotherapy. Patients for whom concurrent chemotherapy is not feasible show limited oncological benefits and PT alone may not be sufficiently effective; therefore, the indication of PT for these patients should be carefully considered. Several studies have demonstrated the efficacy of induction chemotherapy before chemoradiation [31–33]. Induction chemotherapy targets both local tumors and occult metastases and is also considered to have a role in judging tumor biology before treatment [34]. Further studies are warranted to investigate the applicability of induction chemotherapy in PT.

Regarding PT-induced toxicity, the frequency of grade ≥ 3 acute toxicity was 46.5%, with most cases of hematological toxicity thought to be caused by chemotherapy. We had 15 patients (7.5%) with grade ≥ 3 late toxicity gastrointestinal ulcers, including one death due to gastrointestinal perforation. Severe gastrointestinal toxicity can hinder subsequent treatment following PT. Therefore, strict dose limitations in the gastrointestinal tract, regular follow-up, including gastroscopy, and appropriate use of a proton pump inhibitor and mucosal protective agent after PT are crucial.

In the present study, 67.5 GyE PT was administered to all patients. The 1- and 2-year LPFS rates were 84.3% and 68.0%, respectively. These results are comparable with those of other studies on particle radiotherapy for LAPC [8–10]. Recent chemoradiotherapy studies have shown that higher radiation doses result in better outcomes [35–37]. We have reported a new conceptual approach called space-making particle therapy (SMPT), in which a surgical spacer is placed between the tumor and the gastrointestinal tract before PT, SMPT with a Gore-Tex sheet as a spacer for LAPC contributed to significant dose escalation without increasing the dose to the gastrointestinal tract [38]. However, a Gore-Tex sheet is non-bioabsorbable and permanently remains in the patient’s body, possibly causing late complications (e.g., gastrointestinal perforation). Therefore, we use a bioabsorbable spacer made of polyglycolic acid for SMPT to reduce the risk of spacer-related complications [39]. In the future, SMPT for LAPC using bioabsorbable materials may provide significant benefits in terms of long-term survival through dose escalation with less toxicity after PT.

This study had several limitations. This was a single-center retrospective study, and pre-treatment of PT and follow-up were not standardized and were performed at other institutions. In particular, information on the treatment and clinical course after PT is insufficient. In the future, larger multicenter prospective studies are required to confirm the efficacy of PT.

## Conclusion

In summary, our results indicate favorable short- and long-term outcomes after PT for LAPC in the body and tail. SMA invasion, higher pre-PT serum CA 19-9, anterior peripancreatic invasion, and incomplete scheduled concurrent chemotherapy were negative prognostic factors for OS due to worse DMFS. Patients with LAPC in the body and tail with a high risk of distant metastasis may need to rule out potential distant metastases and be combined with systemic chemotherapy.

## Data Availability

The datasets used and analyzed during the current study are available from the corresponding author on reasonable request.

## Abbreviations

CA 19-9: Carbohydrate antigen
CA: Celiac artery
CEA: Carcinoembryonic antigen
CI: Confidence interval
CT: Computed tomography
CTV: Clinical target volume
DMFS: Distant metastasis-free survival
FDG: Fluorodeoxyglucose
GEM: Gemcitabine
GnP: Gemcitabine and nab-paclitaxel
GTV: Gross tumor volume
HR: Hazard ratio
LAPC: Locally advanced pancreatic cancer
LPFS: Local progression-free survival
mOS: Median overall survival
OS: Overall survival
PET: Positron emission tomography
PFS: Progression-free survival
PT: Proton radiotherapy
SMA: Superior mesenteric artery
